# The Relationship between Pain Sensitivity, Pain Catastrophizing and Hangover Severity

**DOI:** 10.3390/ijerph18042047

**Published:** 2021-02-19

**Authors:** Hama M. Saeed, Annabel S. M. Sips, Lauren J. Owen, Joris C. Verster

**Affiliations:** 1 Division of Pharmacology, Utrecht Institute for Pharmaceutical Sciences, Utrecht University, 3584CG Utrecht, The Netherlands; m.saeed@uu.nl (H.M.S.); a.s.m.sips@students.uu.nl (A.S.M.S.); 2 Department of Psychology, School of Health and Society, University of Salford, Frederick Road, Salford M6 6PU, UK; L.J.Owen2@salford.ac.uk; 3 Centre for Human Psychopharmacology, Swinburne University, Melbourne, VIC 3122, Australia

**Keywords:** alcohol, hangover, severity, pain, sensitivity, catastrophizing

## Abstract

Recent research found a significant and positive correlation between hangover severity and pain catastrophizing. The current study aimed to verify these findings. Data from N = 673 subjects with a mean (SD) age of 42.2 (19.1) years old (range: 18 to 87 years old) was evaluated. An online survey collected data on alcohol consumption and hangovers related to their heaviest drinking occasion between 15 January and 14 March 2020. When correcting for the amount of alcohol consumed, significant correlations were found between hangover severity and both sensitivity to pain (r = 0.085, *p* = 0.029) and pain catastrophizing (r = 0.095, *p* = 0.015). In addition, subjective intoxication correlated significantly with sensitivity to pain (r = 0.080, *p* = 0.041) and pain catastrophizing (r = 0.099, *p* = 0.011). Overall, the results were more pronounced in men than women, and the associations with pain catastrophizing were strongest for the subscale assessing rumination. In conclusion, although statistically significant, the observed correlations were of small magnitude. Nevertheless, the observations confirm previous findings that suggest a link between pain perception, alcohol consumption, and hangover severity, which warrants further investigation.

## 1. Introduction

The alcohol hangover has been defined as the combination of negative mental and physical symptoms which can be experienced after a single episode of alcohol consumption, starting when blood alcohol concentration (BAC) approaches zero [1,2]. Although alcohol hangover is the most frequently experienced negative consequence of alcohol consumption [3], scientific knowledge on the pathology of the alcohol hangover is limited [4,5].

Research showed that experiencing (severe) hangovers is not simply a matter of “weakness”: mental resilience scores did not correlate significantly with hangover severity [6]. Over the years, several correlates of hangover frequency and severity have been proposed, including, but not limited to, the amount of alcohol consumed [7,8], congener content [9], and sex [10], but the magnitude of effect that these moderators and mediators is unknown.

An alternative hypothesis may be that drinkers who report more severe hangovers after consuming the same amounts of alcohol may be more sensitive to pain, more preoccupied with pain, or have higher levels of pain catastrophizing. A relationship between experiencing pain and hangovers may be related to overlap in pathology between pain and hangover [11]. For example, in pain processing, the non-opioid morphine metabolite, morphine-3-glucuronide, enhances pain via a Toll-like receptor 4 (TLR4). It appears that in rats TLR4-dependent pain enhancement generalizes to other classes of glucuronide metabolites, which are also present during alcohol metabolism [12]. As such, one could hypothesize that increased sensitivity to pain would correlate closely to sensitivity of experiencing hangover symptoms. However, in humans the relationship between glucuronide and hangover severity has not been established yet, and saliva ethyl glucuronide concentrations showed no significant correlation with hangover severity [13]. Notwithstanding, a substantial number of drinkers (26%) report using analgesic drugs to treat hangovers [14]. Further research is needed to establish the effectiveness of pain medication in the treatment of alcohol hangover.

In addition to a possible overlap in pathology, several typical hangover symptoms concern pain complaints, such as stomach and muscle pain or headache. Especially headache is a frequently reported hangover symptom which can have a significant impact on daily activities [15]. Nevertheless, research on the relationship between pain perception and hangover severity is very limited. Alcohol consumption is an important trigger of delayed headache in both in healthy volunteers [15] and in people who suffer with migraine [16]. The International Headache Society describes hangover headache as “headache caused, after a delay of hours, by ingestion of alcohol (usually in the form of alcoholic beverages)”. The delay of hours is defined as 5–12 h after alcohol consumption (Section 8.1.4.2) [17], similar to the onset of alcohol hangover. Hangover headache is not necessary the same as the common migraine headache. The two types of headache differ in prevalence among men (more often hangover headache) and women (more often migraine headache), and in being experienced unilateral (often in migraine) or bilateral (in hangover) [18].

Research demonstrated that people with migraine are more susceptible to experiencing hangovers [11,19], and therefore they usually limit their alcohol intake to prevent hangover headaches [19]. Migraine-related symptoms such as headache, vomiting, nausea and sensitivity to light and sound are commonly reported symptoms [18], and a direct comparison revealed that migraine patients experience these symptoms more intense than healthy subjects with a hangover [11].

Royle et al. [20] investigated the relationship between pain catastrophizing and hangover severity among N = 86 UK students. They found that hangover severity was significantly and positively correlated with scores on pain catastrophizing. In other words, worrying about pain may increase hangover severity.

In the current study we aimed to replicate and verify the observations by Royle et al. [20]. We further investigated whether there is a relationship of sensitivity to pain per se with reported hangover frequency and severity, and evaluated whether sensitivity to pain and pain catastrophizing is related to subjective intoxication ratings on drinkers’ heaviest drinking occasion. Finally, potential sex differences were explored.

## 2. Materials and Methods

A subsample of alcohol consumers of a large online survey was used for the current analysis [21]. This online survey was conducted between 24 June and 26 July 2020, to collect data on immune fitness, and the psychosocial and health consequences of the COVID-19 pandemic lockdown in Netherlands. Alcohol consumption data was also collected for the period 15 January to 14 March 2020 (i.e., pre-COVID-19 lockdown), and this data was used for the current analysis. The period of assessment was before the start of the COVID-19 pandemic, and public life was therefore not affected by any restrictions. Dutch adults, aged 18 years and older, were invited to participate in the study. The Ethics Committee of the Faculty of Social and Behavioral Sciences of Utrecht University granted ethical approval (approval code: FETC17-061), and electronic informed consent was obtained from all participants.

Questions regarding alcohol consumption were modified from the Quick Drinking Screen for the purpose of this study [22]. For the heaviest drinking occasion within the assessment period, the number of alcoholic drinks consumed as well as the duration of drinking hours (h) were reported. Guidance was provided regarding drinking sizes and how to convert these into units of alcohol. The estimated blood alcohol concentration (BAC) for this occasion was computed using an adapted Widmark equation [23], taking into account sex and body weight. Subjective intoxication (drunkenness) was rated on an 11-point scale ranging from 0 (totally not) to 10 (extremely drunk) [24] and next day hangover severity was assessed, with a range from 0 (no hangover) to 10 (extremely severe) [25]. Participants also reported how many hangovers they had experienced.

Sensitivity to pain was assessed with the Pain Sensitivity Questionnaire (PSQ) [26,27]. For this study, the shortened 10-item version of the PSQ was used [28]. For each item, pain intensity was rated on a scale of 0 (“no pain”) to 10 (“most intense pain imaginable”). The total PSQ score is calculated as the average rating of the 10 items. Higher scores indicate a greater sensitivity to pain. Cronbach’s alpha of the shortened PSQ is 0.91 [28]. Pain catastrophizing was assessed with the shortened Pain Catastrophizing Scale (PCQ) [29]. The scale comprises three questions which can be scored from 1 “not at all” to 5 “always”, addressing the modalities rumination, magnification, and helplessness. A sum score can be computed to serve as measure of pain catastrophizing. Higher scores correspond to greater pain catastrophizing. Cronbach’s alpha of the shortened PCS is 0.892 [29].

Statistical analyses were conducted with SPSS (IBM Corp. Released 2013. IBM SPSS Statistics for Windows, Version 27.0. Armonk, NY, USA). Subjects were included in the analysis when they completed both questions on alcohol consumption and pain. Mean and standard deviation (SD) were computed for all variables. Sex differences were explored using the Independent-Samples Mann–Whitney U test. Differences were regarded statistically significant if *p* < 0.05. Partial correlations, correcting for estimated BAC or the number of alcoholic drinks consumed, were computed between pain outcomes and subjective intoxication, hangover frequency and severity. The analyses were conducted for the full sample, and men and women separately. Finally, alcohol consumption outcomes were correlated with hangover severity and frequency, using Spearman’s correlations. Correlations are considered statistically significant if *p* < 0.05.

## 3. Results

The sample consisted of N = 673 subjects with a mean (SD) age of 42.2 (19.1) years old (range: 18 to 87 years old). A total of 61.4% of the sample were women. Table 1 summarizes their demographics and Table 2 the findings on pain catastrophizing and alcohol consumption mean (SD). Independent comparisons between men and women are presented.

Table 2 shows that compared to men, women consumed significantly less alcohol per week and on their heaviest drinking occasion (*p* < 0.0001) reported experiencing significantly fewer hangovers per month (*p* < 0.0001). No significant sex difference was observed for sensitivity to pain (*p* = 0.087). However, women scored significantly higher on overall pain catastrophizing (*p* < 0.0001), and the individual items rumination (*p* < 0.0001), magnification (*p* = 0.009), and helplessness (*p* = 0.002).

The relationship between pain outcomes, subjective intoxication, and the frequency and severity of alcohol hangover after the heaviest drinking occasion are summarized in Table 3 and Table 4. In Table 3, partial correlations, controlling for the number of alcoholic drinks consumed, are shown. In Table 4, partial correlations, controlling for estimated BAC, are shown.

When conducting the partial correlations controlling for the number of alcoholic drinks (See Table 3), the greatest effect sizes were observed. Overall score for pain catastrophizing and sensitivity to pain correlated significantly with both subjective intoxication and hangover severity positively. No significant correlations with hangover frequency were found. Rumination correlated significantly with subjective intoxication, hangover frequency and severity. Helplessness correlated significantly with subjective intoxication. Other correlations were not significant. For men, significant positive correlations were found between overall pain catastrophizing and hangover severity, rumination and subjective intoxication, rumination and hangover severity, and helplessness and hangover severity. For women, significant positive correlations were found between rumination and subjective intoxication and rumination and hangover frequency.

When controlling for estimated BAC (See Table 4), the correlation analysis revealed no significant correlations of hangover severity and frequency or subjective intoxication with sensitivity to pain or the overall pain catastrophizing score. However, rumination was significantly, positively associated with subjective intoxication and the severity of alcohol hangover. Other variables were not significantly correlated. When conducting the analysis for men only, the correlations between rumination and subjective intoxication and between rumination and hangover severity remained significant. In contrast, in women only one statistically significant correlation was observed between hangover frequency and rumination (See Table 4).

Finally, alcohol consumption outcomes were correlated with hangover severity and frequency. The outcomes are summarized in Table 5 and show that all variables are highly correlated with each other. The strongest predictor of hangover severity and frequency, in both was subjective intoxication. This was observed in both men and women, with no relevant differences in the magnitude of correlations between men and women.

## 4. Discussion

In the current study, significant correlations were found of both sensitivity to pain and pain catastrophizing with hangover severity. Most notable were the significant correlations of rumination with subjective intoxication and the severity and frequency of alcohol hangover. Though, the observed correlations were of small magnitude, they do suggest a link between pain perception and hangover severity. Nevertheless, it is clear that subjective intoxication and other alcohol-related variables have much stronger associations with hangover severity and frequency (see Table 5).

Overall, the results were more pronounced in men than women. However, the magnitude of the differences in correlations was small and thus it can be argued to what extent these sex differences are relevant. Previous research revealed that the magnitude of sex differences in hangover symptom severity were smaller than 1, using the same 11-point scale as in the current study. Van Lawick Pabst et al. [10] concluded that, across different alcohol consumption levels, the sex differences in reported hangover symptom severity were not relevant. A recent meta-analysis revealed that also pain catastrophizing scores assessed with the PCS were unrelated to sex [30].

The results are in line with previous research by Royle et al. [20] and extend these findings by showing that a significant relationship of both sensitivity to pain and pain catastrophizing with hangover severity was also observed for subjective intoxication. There are however differences between the two studies. First, whereas Royle et al. used the original PCS, in the current study the brief 3-item version was used. Additionally, Royle et al. used the Acute Hangover Scale to assess overall hangover severity, whereas the current study used a 1-item scale. Second, whereas Royle et al. included had a small age range and included students only, the current study included all adult age groups including the elderly, with a diversity in employment status (see Table 1). Finally, the current sample size was significantly larger than the Royle et al. sample. The fact that we replicated the findings by Royle et al. in a larger and more diverse sample strengthens the idea that sensitivity to pain and pain catastrophizing are related to hangover severity.

While the data presented in the current study supports a possible relationship between pain and alcohol hangover, more research is needed to further evaluate the impact of pain perception on of hangover severity. Research should also evaluate the relationship of general (non-pain related) rumination, magnification, and helplessness on the severity of alcohol hangover, and investigate the severity of various commonly reported individual hangover symptoms, including those that are less clearly associated with pain, such as sleepiness, concentration problems and apathy. Investigating rumination in general, i.e., beyond pain, is important as increased alcohol consumption and expectancies regarding its (after) effects may be related to specific other, non-pain related, motives for alcohol consumption (e.g., drinking to cope with stress), or expected adverse next-day functional consequences and cognitive impairment that may negatively impact daily activities.

Several limitations of the current study are relevant to mention. First, the data were collected retrospectively. This may have introduced recall bias. Future studies should preferably use a longitudinal design, making assessments in real time, to prevent to possible occurrence of recall bias. Second, shortened versions of pain scales were used to assess sensitivity to pain and pain catastrophizing. While these short versions are validated and reliable, more elaborate versions of these scales available which may be more informative. Third, the data we collected via self-report. Although their subjective nature, the use of validated and reliable scales and questionnaires to assess pain is a common and accepted practice [31]. Whereas research showed that self-reported assessments of sensitivity to heat pain using a simple 1-item assessment (i.e., “Pain doesn’t bother me as much as it does most people”) were not in agreement with actual thermal pain threshold and tolerance experimental assessments [32], studies using the PSQ revealed significant correlations with experimental assessments of pain intensity (r = 0.56, *p* < 0.001), but not to pain thresholds [17]. Given this, it is important to verify the observed relationship between pain sensitivity and hangover severity in experimental studies implementing objective pain threshold tests. It may also be of interest to further investigate the possible role of differences of response styles between subjects [33,34]. Not all individuals are equally capable of sensing and/or expressing their feelings, and this may differ for reporting of psychological and somatic symptoms [34]. Given this, it would be of interest to also investigate the possible role of alexithymia in future research. Further, there may be differences in both alcohol consumption behavior and reporting on pain between countries and individuals with a different cultural and sociodemographic background. Future research should investigate the possible impact of these cross-cultural differences on the observed relationships between pain perception and hangover severity. Fourth, the observed correlations were most pronounced when correcting for the amount of alcohol consumed. When correcting for estimated BAC, the correlations were less strong or became insignificant. It can be argued that correcting for estimated BAC provides more reliable and accurate correlations, better reflecting the true associations between pain perception and alcohol consumption outcomes, as these correlations take into account gender, body weight and dinking duration, in addition to the amount of alcohol consumed. Finally, the magnitude of the correlations was only modest. This suggests that various factors other than pain perception are more important determinants of hangover severity and frequency. These factors may be directly related to alcohol consumption behavior. For example, the amount of alcohol consumed, estimated BAC, and especially subjective intoxication ratings correlated much stronger with hangover severity than pain outcomes.

## 5. Conclusions

While the strength of the observed correlations was modest, the data suggest a relationship between pain perception and hangover severity. Especially rumination was significantly and positively associated with levels of subjective intoxication and the severity of alcohol hangover. Thus, after correcting for estimated BAC, individuals that worry more about pain reported higher levels of intoxication and more severe hangovers. Given the possible overlap in the pathology of pain and alcohol hangover, more research is warranted to further evaluate this relationship.

## Figures and Tables

**Table 1:** Demographics.

Variable	Study Outcome
N	673
Male/Female	260 (38.6%)/413 (61.4%)
Age (years)	42.2 (19.1)
Weight (kg)	78.0 (16.9)
Height (m)	1.74 (0.1)
BMI (kg/m^2^)	25.7 (5.1)
Ethnicity	
Dutch	643 (95.5%)
Migration background	30 (4.5%)
Educational level	
Low	126 (18.7%)
Middle	161 (23.9%)
High	386 (57.4%)
Employment status	
Unemployed	76 (11.3%)
Employer/employee	277 (41.2%)
Student	77 (11.4%)
Student with parttime job	134 (19.9%)
Retired	107 (15.9%)
Not reported	2 (0.3%)

Results for age, weight, height, and BMI are presented as mean (SD); other variables as number (%). Abbreviation: BMI = body mass index, SD = standard deviation.

**Table 2:** Assessments before and during lockdown.

Variables Assessed	Overall	Men	Women
PCS overall	5.2 (2.1)	4.9 (2.0)	5.5 (2.2) *
PCS—Rumination	2.0 (0.8)	1.8 (0.7)	2.0 (0.8) *
PCS—Magnification	1.6 (0.8)	1.5 (0.8)	1.7 (0.9) *
PCS—Helplessness	1.6 (0.9)	1.5 (0.8)	1.7 (0.9) *
PSQ	3.4 (1.6)	3.2 (1.7)	3.4 (1.5)
Alcoholic drinks per week	6.0 (8.6)	9.3 (11.6)	3.8 (4.8) *
Drinking days per week	2.3 (2.1)	3.1 (2.3)	1.9 (1.8) *
Heaviest drinking occasion			
Number of alcoholic drinks	5.9 (5.7)	7.7 (7.1)	4.8 (4.1) *
Drinking duration (h)	4.1 (2.9)	4.4 (3.4)	3.8 (2.6) *
Estimated BAC (%)	0.074 (0.1)	0.078 (0.1)	0.072 (0.1)
Subjective intoxication	2.8 (3.1)	3.0 (3.3)	2.7 (3.0)
Hangover severity	1.7 (2.7)	1.8 (2.7)	1.7 (2.7)
Hangover frequency	0.6 (1.1)	0.7 (1.4)	0.4 (0.9) *

Abbreviations: Pain catastrophizing scale = PCS, Pain sensitivity questionnaire = PSQ, blood alcohol concentration = BAC. Significant sex differences (*p* < 0.05) are indicated by *.

**Table 3:** Relationship of pain perception with subjective intoxication and the frequency and severity of alcohol hangover, when controlling for the number of alcoholic drinks.

Variables Assessed	Subjective Intoxication			Hangover Severity			Hangover Frequency		
	Overall	Men	Women	Overall	Men	Women	Overall	Men	Women
PCS—overall	0.099 *	0.072	0.064	0.095 *	0.149 *	0.002	0.039	0.034	0.044
PCS—Rumination	0.148 ***	0.153 *	0.102 *	0.141 ***	0.231 ***	0.037	0.092 *	0.092	0.099 *
PCS—Magnification	0.014	−0.044	0.012	0.034	0.030	−0.005	−0.024	0.016	0.004
PCS—Helplessness	0.088 *	0.078	0.045	0.068	0.125 *	−0.023	0.033	0.067	0.000
PSQ	0.080 *	0.079	0.052	0.085 *	0.098	0.045	−0.005	−0.037	0.065

Partial correlation values are presented, controlling for the number of alcoholic drinks consumed. Abbreviations: Pain catastrophizing scale = PCS. Pain sensitivity questionnaire = PSQ. Number of asterisk indicate significance level as follows; * <0.05, *** <0.001 (2-tailed).

**Table 4:** Relationship of pain perception with subjective intoxication and the frequency and severity of alcohol hangover, when controlling for estimated BAC.

Variables Assessed	Subjective Intoxication			Hangover Severity			Hangover Frequency		
	Overall	Men	Women	Overall	Men	Women	Overall	Men	Women
PCS—overall	0.044	0.038	0.056	0.054	0.121	0.005	0.002	0.017	0.044
PCS—Rumination	0.112 **	0.146 *	0.100	0.119 **	0.229 ***	0.049	0.071	0.096	0.105 *
PCS—Magnification	−0.037	−0.076	−0.007	−0.006	0.002	−0.019	−0.061	−0.090	0.004
PCS—Helplessness	0.037	0.032	0.047	0.026	0.087	−0.015	−0.002	0.039	0.004
PSQ	0.053	0.082	0.036	0.069	0.107	0.035	−0.005	−0.027	0.056

Partial correlation values are presented, controlling for estimated blood alcohol concentration (BAC). Abbreviations; Pain catastrophizing scale = PCS. Pain sensitivity questionnaire = PSQ. Number of asterisk indicate significance level as follows; * <0.05, ** <0.01, *** <0.001 (2-tailed).

**Table 5:** Relationship of alcohol consumption outcomes and the frequency and severity of alcohol hangover.

Variables	Hangover Severity			Hangover Frequency		
	Overall	Men	Women	Overall	Men	Women
Number of alcoholic drinks	0.662 ***	0.678 ***	0.667 ***	0.632 ***	0.625 ***	0.634 ***
Drinking duration	0.615 ***	0.612 ***	0.617 ***	0.560 ***	0.560 ***	0.558 ***
Estimated BAC	0.583 ***	0.607 ***	0.567 ***	0.546 ***	0.558 ***	0.547 ***
Subjective intoxication	0.805 ***	0.804 ***	0.806 ***	0.740 ***	0.761 ***	0.726 ***

Drinking outcomes for the heaviest drinking occasion were evaluated. Spearman’s r values are presented. Abbreviation: Blood alcohol concentration = BAC. Number of asterisk indicate significance level as follows; *** <0.001 (two-tailed).

## Data Availability

The data of this study is available upon request from the corresponding author.
